# Alternative end-joining mechanisms: a historical perspective

**DOI:** 10.3389/fgene.2013.00048

**Published:** 2013-04-02

**Authors:** Anabelle Decottignies

**Affiliations:** Genetic and Epigenetic Alterations of Genomes, de Duve Institute, Faculty of Pharmacy and Biomedical Sciences, Catholic University of LouvainBrussels, Belgium

**Keywords:** double-strand break, non-homologous end-joining, alternative end-joining, microhomology-mediated end-joining, single-strand annealing, chromosomal translocation, telomere fusion, Ku70/Ku80

## Abstract

In the presence of functional DNA repair pathways, DNA double-strand breaks (DSBs) are mainly repaired by non-homologous end-joining (NHEJ) or homologous recombination (HR), two conserved pathways that protect cells from aberrant chromosomal rearrangements. During the past two decades however, unusual and presumably distinct DNA end-joining repair activities have been unraveled in NHEJ-deficient cells and these are likely to operate in various chromosomal contexts and species. Most alternative DNA end-joining events reported so far appear to involve microhomologous sequences and are likely to rely on a subset of HR enzymes, namely those responsible for the single-strand annealing mechanism of HR, and on DNA Ligase III. Usually, microhomologies are not initially present at DSB ends and thus need to be unmasked through DNA end resection, a process that can lead to extensive nucleotide loss and is therefore highly mutagenic. In addition to microhomology-mediated end-joining events, recent studies in mammalian cells point toward the existence of a distinct and still ill defined alternative end-joining pathway that does not appear to rely on pre-existing microhomologies and may possibly involve DNA Ligase I. Whether dependent on microhomologies or not, alternative DNA end-joining mechanisms are likely to be highly mutagenic *in vivo*, being able to drive telomere fusion events and cancer-associated chromosomal translocations in mouse models. In the future, it will be important to better characterize the genetic requirements of these mutagenic alternative mechanisms of DNA end-joining.

## INTRODUCTION

**FIGURE 1:**
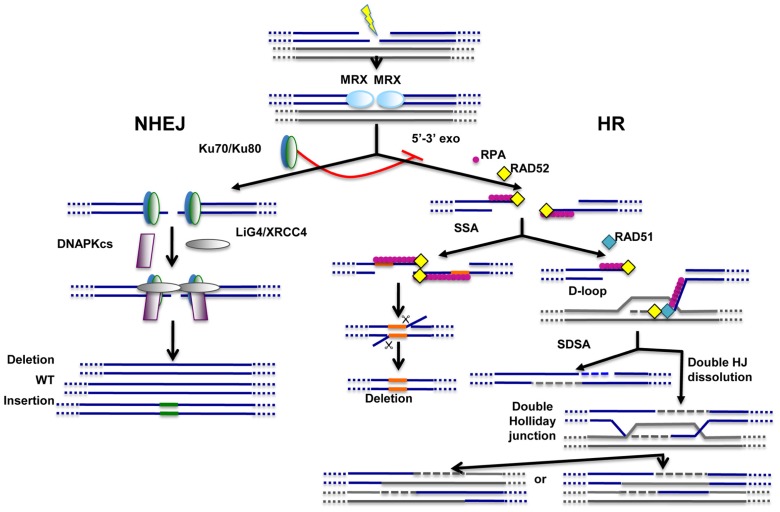
**Main pathways of DNA repair**. Non-homologous end-joining (NHEJ) and homologous recombination (HR) pathways act competitively to repair DNA double-strand breaks (DSBs). Key players of NHEJ and HR are depicted. The MRE11/RAD50/XRS2 (MRX) complex is recruited very early at DNA ends and appears to play important roles for both NHEJ and HR. Ku70/Ku80 heterodimer is required for NHEJ and, through inhibition of DNA end resection (5′–3′ exo), acts as a repressor of HR. Fidelity of NHEJ-dependent DSB repair is low and, most of the time, associated with nucleotide deletions and/or insertions at repair junctions. The common early step of HR-dependent mechanisms is the formation of ssDNA which is then coated by replication protein A (RPA). Single-strand annealing (SSA) mechanism requires the presence of direct repeats (shown in orange) on both sides of the break. SSA does not imply any strand invasion process and is therefore not dependent on RAD51 protein. Strand invasion and D-loop formation are however common steps of synthesis-dependent strand annealing (SDSA) and double Holliday junction (HJ) dissolution mechanisms. In the latter case, double Holliday junctions are resolved with or without crossing-over.

Double-strand breaks (DSBs) represent major threats to genome integrity. They can be induced during normal metabolism or may result from the presence of exogenous genotoxic agents like ionizing radiations or chemotherapeutic drugs. Cells have evolved two main pathways to repair these lesions: the non-homologous end-joining (NHEJ) pathway, that ensures direct resealing of DNA ends; and the homologous recombination (HR) pathway that relies on the presence of homologous DNA sequences for DSB repair. Repair through HR is not defined by a unique mechanism but operates through various mechanistically distinct DSB repair processes including synthesis-dependent strand annealing (SDSA), double Holliday junction resolution, and single-strand annealing (SSA; Paques and Haber, 1999; Chapman et al., 2012; **Figure 1**). The common step for HR-dependent DSB repair mechanisms is the initial formation of single-stranded DNA (ssDNA) for pairing with homologous DNA template sequences. These HR-dependent mechanisms of DSB repair have been extensively reviewed previously and will not be detailed here (Paques and Haber, 1999; Chapman et al., 2012).

Until very recently, how cells choose between NHEJ and HR-dependent pathways for DSB repair was still unclear, although both the cell cycle stage and the nature of DSB ends were previously involved (Symington and Gautier, 2011; Chapman et al., 2012). A critical determinant for the choice is provided by the 5′–3′ resection of DNA ends that, while triggering HR-dependent repair, prevents NHEJ. On the contrary, direct binding at DSB ends of the conserved Ku70/Ku80 heterodimer, a key complex of the NHEJ pathway that protects DNA ends against exonucleases, represses HR-dependent mechanisms (Pierce et al., 2001). Four studies recently shed new light on this important question of initial choice between NHEJ and HR for DSB repair and provided consistent evidences in favor of the existence of a tightly regulated mechanism (Chapman et al., 2013; Di Virgilio et al., 2013; Escribano-Diaz et al., 2013; Zimmermann et al., 2013). Four proteins were shown to play critical roles in repair pathway choice: RIF1 (Rap1-interacting factor 1), 53BP1 (p53 binding protein 1), BRCA1 (breast cancer type 1 susceptibility protein), and CtIP (C terminus-binding protein-interacting protein). Briefly, it appears that, while 53BP1–RIF1 stimulates NHEJ, BRCA1 and CtIP together promote DNA end resection and HR. RIF1 is indeed recruited to DSB ends via an interaction with 53BP1 and both proteins cooperate to promote NHEJ in G1 cells. During G2, RIF1/53BP1 binding to DNA ends is repressed by BRCA1, ensuring a switch to HR during this stage of the cell cycle. Collectively, these studies provide strong evidences in favor of well-regulated competitions between NHEJ and HR pathways at DNA ends.

The choice of DSB repair pathway inevitably impacts on the fidelity of repair. Indeed, while HR is generally viewed as a conservative DSB repair pathway, NHEJ operates with poor fidelity and nucleotide deletions and/or insertions are frequently detected at repair junctions (**Figure 1**). However, not all HR-dependent mechanisms display high fidelity of repair. Namely, the SSA mechanism requires annealing at two directly repeated DNA sequences near DSB ends. Consequently, intervening nucleotides initially present between the direct repeat sequences flanking the break are lost during SSA-mediated repair (**Figure 1**).

More recently, backup pathways for DNA repair were identified in NHEJ-deficient cells of various organisms. Although it is still unclear whether only one or several backup repair pathways exist, they do not rely on large homologous DNA templates such as those involved in HR-dependent repair events and are therefore referred to as “alternative end-joining” pathways. However, alternative end-joining mechanisms usually -but not always- rely on the presence of microhomologies at or near DSB ends, suggesting that these repair events may not be entirely distinct from HR-dependent mechanisms. Here, I review the history of alternative end-joining discovery and the recent evidences that these alternative end-joining events are able to drive class switch recombination in the immune system, telomere fusions and chromosomal translocations *in vivo.*

## ALTERNATIVE END-JOINING REPAIR: ONE OR MORE BACKUP PATHWAY(S)?

Proteins required for NHEJ include -but are not restricted to- the highly conserved Ku70/Ku80 heterodimeric complex, DNA-dependent protein kinase catalytic subunit (DNA-PKcs) and DNA Ligase IV (LIG4) in complex with XRCC4 (Weterings and Chen, 2008). By directly binding DNA ends, Ku70/Ku80 ensures protection against exonucleases and, as such, acts as an inhibitor of HR (**Figure 1**). In 1996, thanks to the use of Ku70-deficient budding yeast mutants, Boulton and Jackson provided the first evidences for the existence of an alternative DNA end-joining pathway. This pathway was about 20-fold less efficient than NHEJ and repair junctions displayed both nucleotide deletions and overlapping microhomologies of 3–16 nucleotides (Boulton and Jackson, 1996). Although it was known at that time that short microhomologous regions of up to five nucleotides were commonly recovered at NHEJ repair junctions of mammalian cells (Roth et al., 1985), this DNA repair pathway was clearly able to operate in a NHEJ-deficient background. Supporting the existence of a new DNA end-joining pathway, biochemical fractionation of calf thymus extracts yielded two fractions with distinct DSB repair activities (Mason et al., 1996). One fraction, presumably enriched for microhomology-mediated end-joining (MMEJ) activity, was clearly relying on the presence, on both sides of the DSB, of short repeat sequences. The second fraction, containing the NHEJ activity, was characterized by the presence of a DNA fill-in activity -inhibited by DNA polymerase inhibitors- and the ability to perform ligation of non-homologous DNA fragments (Mason et al., 1996). In agreement with the previous suggestion that very short sequence homologies are likely to help DNA end alignment prior to NHEJ-dependent repair (Roth et al., 1985; Roth and Wilson, 1986), some repair junctions produced by the NHEJ activity-containing fraction were also characterized by the presence of overlapping microhomologies. However, the remaining repair junctions were devoid of any microhomology, indicating that microhomologies were not strictly required for NHEJ in this system (Mason et al., 1996). Later on, backup pathways of end-joining were identified in various NHEJ-deficient mammalian cells (Kabotyanski et al., 1998; Feldmann et al., 2000; Wang et al., 2003).

Whether this newly identified backup MMEJ pathway was involving a new set of DNA repair proteins was unclear at that time. In 1994, and although they were working in a NHEJ-proficient budding yeast background, the group of Haber first postulated that microhomology-mediated DNA repair events may occur through a RAD52-dependent SSA-type mechanism (Kramer et al., 1994). The same group then reported that the lower limit for SSA-dependent DSB repair was lying between 5 and 29 bp of homology, showing that sequence homologies may be very low for HR, at least in budding yeast (Sugawara et al., 2000). They suggested however, that some differences may exist between classical SSA, involving large direct repeats, and “micro-SSA”, in which homology lengths are much lower, as the latter process appeared to rely mostly on RAD59, a budding yeast homolog of RAD52, instead of RAD52 itself (Sugawara et al., 2000). Altogether, studies by the group of Haber thus pointed toward the possible contribution of HR-dependent pathways in budding yeast MMEJ, suggesting that this may not represent a new completely distinct DNA repair pathway but could reflect a micro-SSA-type mechanism of DSB repair. In complete agreement with these predictions, a study performed in *X. laevis *eggs established that a purified fraction displaying MMEJ activity contained DNA Ligase III (LIG3), DNA polymerase ε, FEN-1 endonuclease, and exonuclease activities of 5′–3′ and 3′–5′ directionality and that the same fraction was able to process SSA intermediates (Gottlich et al., 1998). Next, it was reported that, in a NHEJ-deficient *S. cerevisiae *background, MMEJ events were not dependent on RAD52 but required the MRE11/RAD50/XRS2 complex previously implicated in both NHEJ and HR (Ma et al., 2003; Yu and Gabriel, 2003). The requirement for RAD59 was however, not tested. Extrachromosomal DSB repair experiments in NHEJ-deficient fission yeast mutants then provided additional evidences in favor of a SSA-dependent mechanism for MMEJ (Decottignies, 2007). In this system, both RAD22, the fission yeast RAD52 homolog, and EXOl, the 5′–3′ exonuclease involved in the formation of ssDNA intermediates for HR, were required for MMEJ (Decottignies, 2007). However, another study conducted in mouse ES cells concluded that, although the first steps may be shared, alternative NHEJ in ES cells may be distinct from SSA during the late steps of repair (Bennardo et al., 2008). This conclusion came from the observation that mouse RAD52 was not able to stimulate alternative NHEJ in their experimental chromosomal context although the protein was able to promote SSA when the entire coding sequence of GFP was involved in homology-directed repair (Bennardo et al., 2008). One possibility however, would be that the annealing process may require another protein than RAD52, similarly, to the situation in budding yeast where RAD59, a RAD52 homolog, is required for annealing when only very short homologous sequences are available for SSA (Sugawara et al., 2000). In support of this, observations in *RAD52 *knock-out mouse models suggested that mouse RAD52 may only be involved in certain types of DSB repair processes while other HR-dependent events may be catalyzed by distinct proteins functionally related to RAD52 (Rijkers et al., 1998). This remains to be tested experimentally.

Additional proteins, whether from yeast or from higher eukaryotes, were reported to play a role in MMEJ. POL4, a member of the PolX family of polymerases with gap-filling activity, and proteins from the mismatch repair pathways were found to be required for MMEJ-dependent repair of substrates with non-perfect microhomologies in fission yeast (Decottignies, 2007). The MRE11 complex was found to be required for MMEJ in budding yeast (Ma et al., 2003), *Arabidopsis *(Heacock et al., 2004) and human cells (Delia-Maria et al., 2011), but dispensable for fission yeast MMEJ using an extrachromosomal DSB repair assay (Decottignies, 2007). It is believed however, that fission yeast MRE11 complex may be required for MMEJ events in a chromosomal context and/or for intermolecular MMEJ-dependent ligations (Decottignies, 2005, 2007). As stated above, first evidences for the involvement of LIG3 in the MMEJ process were provided by biochemical fractionation of *X. laevis *egg extracts (Gottlich et al., 1998). LIG3 contribution to MMEJ was later confirmed in HeLa cells (Wang et al., 2005), in human HTD114 cell line (Liang et al., 2008) and in mice (Simsek et al., 2011).

In mature mouse B cells activated by antigens, recent *in vivo *evidences indeed support the existence of a powerful backup mechanism able to compensate for NHEJ during immunoglobulin class switch recombination (CSR; Soulas-Sprauel et al., 2007; Yan et al., 2007). Whether this *in vivo *backup mechanism is similar to the MMEJ repair pathway described above is however still a matter of debate. In favor of this hypothesis, the backup CSR activity detected in the absence of either XRCC4 or LIG4 was found to operate through the recognition of microhomologies at DNA break borders and, in agreement with two previous reports (Audebert et al., 2004; Wang et al., 2005), was proposed to rely on XRCC1/LIG3 complex (Yan et al., 2007). Interestingly, XRCC1 was previously involved in SSA (Stark et al., 2004), further supporting the view that alternative NHEJ may similarly operate through a micro-SSA-like mechanism in immune cells. A more recent study published by the group of Jasin reported that, similarly to what happens in human cells in culture, mouse LIG3 is involved in an alternative end-joining pathway operating through annealing at pre-existing microhomologies (Simsek et al., 2011). They proposed that LIG4 was acting as a repressor of the DNA end resection activity required to produce the complementary ssDNA ends.

Although the studies reported above in various eukaryotic species converged onto the identification of the alternative end-joining backup pathway as a microhomology-dependent mechanism presumably relying on LIG3, recent data led to revise this view. Indeed, experiments performed in mammalian cells suggested the existence of an additional alternative end-joining pathway presumably relying on Ligase I (LIG1) and able to repair DSBs independently of pre-existing microhomologies (Boboila et al., 2010a,b, 2012; Simsek et al., 2011). First *in vivo *evidences came from the observation that, in either *KU70*^-/-^ or *KU70*^-/-^/LIG4^-/-^ mice, CSR appears to operate through two distinct alternative end-joining mechanisms in B cells, with only one relying on microhomologies (Boboila et al., 2010a). This newly unraveled alternative end-joining mechanism was not initially detected in either *LIG4*^-/-^ or *XRCC4*^-/-^ mouse B cells for which microhomologies were recovered at all CSR junctions, suggesting that it may be repressed by Ku70/Ku80. Further support in favor of the existence of a second alternative end-joining mechanism not relying on pre-existing microhomologies was provided by sequencing of chromosomal translocation breakpoints recovered in B cells from *KU70*^-/-^ mice (Boboila et al., 2010b). Shortly after, using an experimental system of chromosomal translocation induction based on zinc finger nuclease-induced DSBs in mouse cells, the group of Jasin reported that, while translocation breakpoints displayed less microhomologies in the absence of LIG3, LIG1 depletion did not affect microhomology use (Simsek et al., 2011). Note however that an *in vitro *study performed in human HTD114 cell line reported that both LIG1 and LIG3 were involved in MMEJ-dependent repair of an extrachromosomal DSB, although contribution of LIG3 appeared to be more important (Liang et al., 2008).

**FIGURE 2:**
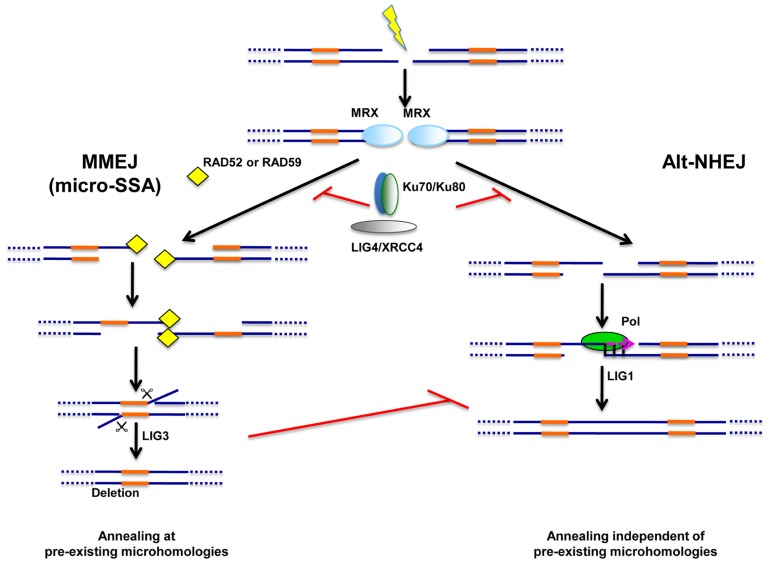
**Alternative end-joining pathways of DNA repair**. Two types of alternative end-joining pathways of DSB repair were unraveled in NHEJ-deficient cells. One pathway, dubbed “microhomology-mediated end-joining” or MMEJ, relies on pre-existing microhomologies around the break (in orange) and is likely to operate through a mechanism related to single strand-annealing (micro-SSA). MMEJ appears to rely on Ligase III (LIG3) for sealing. Unlike MMEJ, the second alternative pathway, dubbed Alt-NHEJ, does not require the presence of pre-existing microhomologies and may rather rely on Ligase I (LIG1). Simsek et al. (2011) proposed that microhomologies may nevertheless be generated by a polymerase activity (Pol) operating at one DNA end. The same study suggested that LIG1 may only function in the absence of LIG3 as a back-up ligase, at least in mouse cells. Both MMEJ and Alt-NHEJ are repressed by the NHEJ machinery (Ku70/Ku80, LIG4/XRCC4). The MRX complex is likely to play important roles for both alternative pathways during the first steps of repair.

Altogether, data suggest the possible existence of two distinct alternative end-joining repair processes, both repressed by Ku70/Ku80 (**Figure 2**), The first one appears to rely on the presence of microhomologies for repair and I propose that it operates through a micro-SSA-type mechanism and involves LIG3. The second pathway of alternative end-joining does not appear to depend on pre-existing microhomologies and is believed to rely on LIG1. However, evidences for the conservation of the latter pathway throughout the eukaryotic lineage are still lacking.

## IMPLICATION OF ALTERNATIVE NHEJ IN TELOMERE FUSION EVENTS

Telomeres at the end of linear chromosomes are reportedly resistant to end-to-end fusions thanks to the binding of so-called shelterin proteins (van Steensel et al., 1998). Accordingly, when normal shelterin density is breached, protection of telomeres against DNA damage activation is no longer ensured and DNA repair enzymes have access to telomeric DNA. Hence, loss of TRF2 shelterin component at mammalian telomeres induces telomere deprotection and LIG4-dependent fusions (Smogorzewska and de Lange, 2002). Further experiments revealed a direct role for mammalian RAP1/TRF2 complex in protection of telomeric DNA from NHEJ, independently of the involvement of TRF2 in telomeric-loop formation (Bae and Baumann, 2007).

Although NHEJ is clearly able to mediate end-to-end fusions in a telomere-deficient background, this DNA repair pathway is not required to catalyze all types of telomere fusions. Indeed, telomerase-deficient fission yeast mutants lacking either Ku70 or LIG4 display rearranged telomeres and chromosome circularization, indicating that alternative end-joining mechanisms are able to promote telomere fusion (Baumann and Cech, 2000). Similar conclusions were subsequently drawn from studies in budding yeast (Mieczkowski et al., 2003) and *Arabidopsis *(Riha and Shippen, 2003). A molecular analysis was then published in which authors analyzed telomere fusion events in *Arabidopsis *mutants lacking both TERT catalytic subunit of telomerase and Ku70 DNA repair protein (Heacock et al., 2004). Fusions between telomeric and subtelomeric regions of plant chromosomes were associated with large deletions, extending to more than 300 bp, and displayed overlapping homologies of up to 12 bp. Here too, Ku70 was acting as a strong inhibitor of the MMEJ-dependent mechanism of telomere fusion while MREll was found to promote fusions (Heacock et al., 2004). Subsequent work by the same group revealed that, as expected from an alternative end-joining mechanism, LIG4 was not required for plant telomere fusions (Heacock et al., 2007). Studies in human cells revealed similar mechanisms of telomere fusion in cells forced to divide in the absence of telomerase. In these cells, telomere fusions occurred with concomitant deletion of one or both telomeres and were characterized by the presence of microhomologies (Capper et al., 2007; Letsolo et al., 2010).

Following these *in vitro *studies, a report provided evidences for the involvement of both NHEJ and MMEJ repair pathways in mouse telomere fusion events *in vivo *(Rai et al., 2010). Using a combination of mutants and shRNA constructs, the authors showed that, while TRF2/RAP1 complex protects telomeres from ATM activation and NHEJ, single-stranded telomeric DNA-binding protein POT1, in conjunction with TPP1 shelterin component, inhibits ATR activation and alternative NHEJ. In agreement with previous data in human cells, their work further suggested that alternative NHEJ is the main pathway to process dysfunctional telomeres in mouse cells experiencing natural telomere erosion (Rai et al., 2010). Hence, despite a strong protection against NHEJ provided by TRF2, mammalian telomeres can be targets of MMEJ. Elegant demonstration of the role of shelterin components in telomere end protection was recently provided by the group of de Lange (Sfeir and de Lange, 2012). They confirmed the role of TRF2 as repressor of both ATM signaling and classical NHEJ and the role of POT1 in ATR signaling repression. They also showed that alternative NHEJ was repressed by various shelterin components as well as by Ku70/Ku80 and proposed that the redundancy of repressors may ensure better protection against dangerous alternative NHEJ at telomeres (Sfeir and de Lange, 2012).

Hence, the above studies clearly pointed toward an important contribution of alternative NHEJ to pathologic chromosome fusion events in cells with dysfunctional telomeres. Although evidences for an involvement of SSA proteins in these end-joining events is still lacking, telomere fusions are characterized by microhomologies at junctions, are repressed by Ku70/Ku80 and rely on the MRE11 complex in plants and possibly also in human cells (Tankimanova et al., 2012).

## MUTAGENIC POTENTIAL OF ALTERNATIVE NHEJ IN MAMMALS

In the late 1990s, it became evident that NHEJ acts as a tumor suppressing mechanism. Indeed, mice lacking both p53 and a NHEJ component, like DNA-PKcs, Ku80, XRCC4, or LIG4, were found to die in early postnatal life due to an elevated frequency of B cell lymphomas displaying *IgH-Myc *translocations and amplifications (reviewed in Sharpless et al., 2001). Importantly, these lymphomas were qualitatively distinct from those arising in a p53-deficient background alone as, in the latter mouse mutants, tumors had a later onset and did not generally harbor translocations. Following these observations, *IgH-Myc *translocation junctions were recovered from *XRCC4*^-/-^/*p53*^-/-^ mice in order to characterize the DNA repair mechanisms involved in chromosomal translocations. Sequencing of breakpoint junctions revealed the presence of microhomologous DNA sequences (Zhu et al., 2002; Wang et al., 2008). *LIG4 *haploinsufficiency was also reported to increase sarcoma formation in *INK4a/ARF*^-/-^ mice by inducing chromosomal translocations, amplifications and deletions but translocation junctions were not characterized (Sharpless et al., 2001). In a more recent report, the group of F. Alt confirmed that an alternative end-joining pathway robustly catalyzes translocations in *KU70*^-/-^/*LIG4*^-/-^ mice B cells that are fully deficient for classical NHEJ (Boboila et al., 2010b). However, as the authors did not detect any bias toward MMEJ at breakpoint junctions, they suggested that translocations were mediated by an alternative end-joining mechanism not relying on microhomologies. It should be tested whether this mutagenic alternative end-joining mechanism operating in B cells of *KU70*^-/-^ mice requires LIG1.

In human, an analysis of high-grade bladder carcinomas suggested that MMEJ may contribute to the high genomic instability of bladder cancer (Bentley et al., 2004). Indeed, authors showed that these tumors were highly proficient in their ability to perform MMEJ, even though Ku, DNA-PKcs and XRCC4 proteins were expressed at normal level.

Altogether, data reported so far indicate that, although the classical LIG4/Ku-dependent NHEJ pathway appears to act as a potent tumor suppressor mechanism, alternative end-joining pathways, whether relying on microhomologies or not, promote chromosomal translocations. In the future, it would be interesting to better characterize these alternative pathways of end-joining and to identify the genes involved in the repair processes.

## Conflict of Interest Statement

The author declares that the research was conducted in the absence of any commercial or financial relationships that could be construed as a potential conflict of interest.
